# Resource: A multi‐species multi‐timepoint transcriptome database and webpage for the pineal gland and retina

**DOI:** 10.1111/jpi.12673

**Published:** 2020-07-08

**Authors:** Eric Chang, Cong Fu, Steven L. Coon, Shahar Alon, Marjan Bozinoski, Matthew Breymaier, Diego M. Bustos, Samuel J. Clokie, Yoav Gothilf, Caroline Esnault, P. Michael Iuvone, Christopher E. Mason, Margaret J. Ochocinska, Adi Tovin, Charles Wang, Pinxian Xu, Jinhang Zhu, Ryan Dale, David C. Klein

**Affiliations:** ^1^ Bioinformatics and Scientific Programming Core Eunice Kennedy Shriver National Institute of Child Health and Human Development National Institutes of Health Bethesda MD USA; ^2^ Section on Neuroendocrinology Program in Developmental Endocrinology and Genetics Eunice Shriver Kennedy National Institute of Child Health and Human Development National Institutes of Health Bethesda MD USA; ^3^ Key Laboratory of Organ Regeneration & Transplantation of the Ministry of Education The First Hospital of Jilin University Changchun China; ^4^ Laboratory of Theoretical and Computational Chemistry Institute of Theoretical Chemistry Jilin University Changchun China; ^5^ National‐Local Joint Engineering Laboratory of Animal Models for Human Diseases Changchun China; ^6^ Molecular Genomics Core Office of the Scientific Director Eunice Kennedy Shriver National Institute of Child Health and Human Development National Institutes of Health Bethesda MD USA; ^7^ Department of Neurobiology The George S. Wise Faculty of Life Sciences, and Sagol School of Neuroscience Tel‐Aviv University Tel Aviv Israel; ^8^ Department of Physiology and Biophysics and the Institute for Computational Biomedicine Weill Cornell Medical College New York NY USA; ^9^ Computer Support Services Core Eunice Shriver Kennedy National Institute of Child Health and Human Development National Institutes of Health Bethesda MD USA; ^10^ Departments of Ophthalmology and Pharmacology & Chemical Biology Emory University School of Medicine Atlanta GA USA; ^11^ Center for Genomics School of Medicine Loma Linda University Loma Linda CA USA; ^12^ Department of Genetics and Genomic Sciences Mount Sinai School of Medicine Icahn Medical Institute New York NY USA; ^13^ United States Food and Drug Administration’s National Center for Toxicological Research, Food and Drug Administration Jefferson AR USA; ^14^ Department of Physiology School of Basic Medical Sciences Anhui Medical University Hefei China; ^15^ Office of the Scientific Director Eunice Kennedy Shriver National Institute of Child Health and Human Development National Institutes of Health Bethesda MD USA; ^16^ Present address: The Alexander Kofkin Faculty of Engineering Bar‐Ilan University Ramat‐Gan Israel; ^17^ Present address: Instituto de Histología y Embriología de Mendoza Consejo Nacional de Investigaciones Científicas y Técnicas Mendoza Argentina; ^18^ Present address: West Midlands Regional Genetics Laboratories Birmingham, Women’s and Children’s NHS Foundation Trust Birmingham UK; ^19^ Present address: National Heart, Lung and Blood Institute National Institutes of Health Bethesda MD USA; ^20^ Present address: The Faculty of Life Sciences Bar‐Ilan University Ramat‐Gan Israel

**Keywords:** biological rhythms, neurotranscriptomics, pineal, retina, RNA‐Seq, transcriptome, webpage

## Abstract

The website and database https://snengs.nichd.nih.gov provides RNA sequencing data from multi‐species analysis of the pineal glands from zebrafish (*Danio rerio*), chicken (White Leghorn), rat (*Rattus nove* *gicus*), mouse (*Mus musculus*), rhesus macaque (*Macaca mulatta*), and human (*Homo sapiens*); in most cases, retinal data are also included along with results of the analysis of a mixture of RNA from tissues. Studies cover day and night conditions; in addition, a time series over multiple hours, a developmental time series and pharmacological experiments on rats are included. The data have been uniformly re‐processed using the latest methods and assemblies to allow for comparisons between experiments and to reduce processing differences. The website presents search functionality, graphical representations, Excel tables, and track hubs of all data for detailed visualization in the UCSC Genome Browser. As more data are collected from investigators and improved genomes become available in the future, the website will be updated. This database is in the public domain and elements can be reproduced by citing the URL and this report. This effort makes the results of 21st century transcriptome profiling widely available in a user‐friendly format that is expected to broadly influence pineal research.

## 1 INTRODUCTION

The pineal transcriptome has been studied for over 30 years, starting with Northern blot detection of single transcripts encoding proteins involved in melatonin synthesis, including those encoding Tph1 and Asmt (Hiomt).^1^, ^2^, ^3^, ^4^ Since then, pineal transcriptomics has spanned the development of transcriptomic assays including cDNA‐based hybridization technology, qRT‐PCR, and RNA‐Seq.^5^, ^6^, ^7^, ^8^, ^9^, ^10^, ^11^, ^12^, ^13^, ^14^, ^15^

High‐throughput sequencing offers many advantages for assaying the transcriptome, but the field of bioinformatics moves quickly with tools, algorithms, and genome assemblies changing from year to year. As a result, data from earlier studies cannot be meaningfully compared with data from later studies that used different methods without completely re‐processing all data uniformly using updated methods, assemblies, and annotations. This has been recognized for example in the recount2 project,^16^ which has re‐processed tens of thousands of human RNA‐Seq samples from public repositories uniformly.

Furthermore, for detailed study of particular loci it is critical to visualize expression alongside genomic data from other studies. Genome browsers such as the UCSC Genome Browser^17^ allow just this. In particular, this browser supports track hubs that allow for the configuration, coloration, and organization of collections of many tracks using a web interface.^18^ This allows researchers to generate highly customized views tailored to their research interest, viewing pineal gland data from this study directly alongside a wealth of publicly available data prepared and made available by the UCSC team.

Cross‐species transcriptome reports appear in the literature, focused on two‐species comparisons, for example, mouse versus human^19^ and zebrafish versus human.^20^ Here, we introduce a website that aggregates multiple RNA‐Seq studies of the pineal gland spanning years of transcriptomics research on six species across three vertebrate classes, processed uniformly and presented in a user‐friendly site allowing inspection of individual genes as well as UCSC Genome Browser track hubs of each experiment. The site can be found at https://snengs.nichd.nih.gov. The results of this effort facilitate the comparative and evolutionary analysis of the pineal gland and retina, reflecting an interest in the evolutionary history that links these tissues as derivatives of a common ancestral photodetector.^21^, ^22^ Most of the tens of thousands of transcripts profiled are otherwise absent from the pineal and retinal literature and in many cases have not been well studied in any tissue. Accordingly, the web page opens new avenues of research.

This report alerts investigators to the availability of this resource, which will be of special value where user‐friendly compiled pineal and retinal RNA‐Seq data are otherwise not available in any format. The graphs and other information extracted from the web page are in the public domain. The web page and its underlying infrastructure is designed to be easily updated as data from new experiments become available or as reanalysis of existing datasets using improved software and updated genomes is completed.

## 2 METHODS

### 2.1 Animals

Samples were collected to identify differential day/night ratios; and, in the case of the rat, expression was studied as a function of development, denervation, and adrenergic–cyclic AMP stimulation (Table 1).^9^, ^12^, ^23^, ^24^, ^25^ In many cases, retinal tissue was profiled in parallel. Mixed tissue RNA samples were used in conjunction with the pineal gland and retina to estimate the enrichment of a transcript.

**Table 1:** Experiments on the database

Study No.	Animals	Experiment name	Tissue	Lighting	Sampling times	Notes	Reps	Refs
101	Chicken, White Leghorn	Pineal gland and retina; time series; constant darkness	PG, R	D:D	CT 0, 4, 8, 12, 16, 20	N/A	3	N/A
102	Human	Pineal gland; day and night	PG	L:D 12:12	ZT 6, 18	N/A	2, 4	N/A
103	Mouse, 129sv	Pineal gland, retina and mixed tissue; day and night; Eya2 KO	PG, R, MT	L:D 12:12	ZT 6, 18	Eya2 KO	1	N/A
104	Rat, Sprague Dawley	Pineal gland; day and night; and mixed tissue, day; polyA	PG, MT	L:D 14:10	ZT 7, 19	N/A	1	23
105	Rat, Sprague Dawley	Pineal gland development; day and night	PG	L:D 14:10	ZT 7, 19	Ages: E21, P5, P20, P40	1	23, 24
106	Rat, Sprague Dawley	Pineal gland (RP), retina (RR) and mixed tissue (RX); 24‐hr time series	PG, R, MT	L:D 14:10	ZT 1, 7, 13, 15, 19, 23	N/A	1	23
107	Rat, Sprague Dawley	Pineal gland; day and night; and mixed tissue, day; Ribominus	PG, MT	L:D 14:10	ZT 7, 19	N/A	1	23
108	Rat, Sprague Dawley	Pineal gland; superior cervical decentralization (DCN) or ganglionectomy (SCGX); day and night	PG	L:D 14:10	ZT 7, 19	DCN, SCGX, Sham, Control	3	8
109	Rat, Sprague Dawley	Pineal gland in vitro; norepinephrine (NE) or dibutyryl cyclic AMP (DBcAMP)	PG	N/A	N/A	Cultured glands; NE, DBcAMP, Control	3	8
110	Rat, Sprague Dawley	Pineal gland marker genes; day and night	PG	L:D 14:10	ZT 7, 19	N/A	1	23
111	Rhesus macaque	Pineal gland, retina and mixed tissue; time series	PG, R, MT	L:D 12:12	ZT 6, 12, 18, 24	Dawn, Day, Dusk, Night	3	12
112	Zebrafish	Pineal gland; time series; constant darkness	PG	D:D	CT 2, 6, 10, 14, 18, 22	N/A	2	7
113	Zebrafish	Eye, pineal gland and mixed tissue; day and night	Eye, PG, MT	L:D 12:12	ZT 6, 18	clocka KO	1	N/A

Note

Thirteen experiments encompassing six species are on the website; additional experiments are to be added as data become available. Experimental details are available on the website (https://snengs.nichd.nih.gov/experiments) and the listed references.

Abbreviations: CT, circadian time; D:D, constant darkness; L:D, light:dark; MT, mixed tissue; N/A, not available; PG, pineal gland; R, retina; Refs, references; Reps, number of replicates; RP, rat pineal gland; RR, rat retina; RX rat mixed tissue; ZT, Zeitgeber time.

### 2.2 Sequencing

Illumina sequencing was used in all cases. Specific experimental details are available on each experiment's page on the website (see https://snengs.nichd.nih.gov/experiments) that recapitulates original experimental methods in the respective original manuscript. All data across all species were re‐analyzed in the assemblies and annotations described (https://snengs.nichd.nih.gov/methods). Quality of FASTQ files was analyzed with FastQC v0.11.8 and MultiQC v1.6 and all samples demonstrated high‐quality sequencing. Adapters were removed and light quality trimming was performed with cutadapt v1.18 using additional arguments ‐q‐minimum length 25.

These trimmed reads were provided to Salmon v0.12.0^26^ for transcript quantification using an index built for transcriptomes as described (https://snengs.nichd.nih.gov/methods), and run using the additional arguments –gcBias –seqBias ‐‐libTypeA. For each gene, the per‐transcript values reported by Salmon v0.12.0 were summed to provide a gene‐level expression estimate in units of transcripts per million reads (TPM). These are the values reported in the tables and plots of each gene page.

### 2.3 Genomic visualization

For genomic visualization in the UCSC Genome Browser, trimmed reads were aligned using HISAT2 v2.1.0 to the respective genome indicated below. From these aligned reads, normalized bigWig files were created using the deepTools v3.1.3 bamCoverage tool using additional options ‐‐minMappingQuality 20 ‐‐smoothLength 10 ‐‐normalizeUsing BPM ‐‐binSize 1 such that multi‐mappers were ignored. For stranded libraries, the tool was run twice: once with ‐‐filterRNAstrand forward and once with ‐‐filterRNAstrand reverse to get separate tracks for each strand. The resulting bigWig files were combined into a UCSC track hub using the trackhub Python package.

UCSC (typically used for extensive visualization capabilities) and Ensembl (typically used for its comprehensive annotations) are not consistent in their chromosome nomenclature. To facilitate linking from gene‐level transcription estimates on this website to genomic signal at UCSC, we converted chromosome names from Ensembl to the UCSC equivalents by matching the md5sums of each chromosome; see GitHub repository (https://github.com/NICHD‐BSPC/chrom‐name‐mappings) for details and code.

### 2.4 Genome and transcriptome assemblies

For each species, the genomic assembly indicated (https://snengs.nichd.nih.gov/methods) was used for visualization in the UCSC Genome Browser, while the transcriptome was used to calculate TPM expression estimates to display in plots on individual gene pages.

### 2.5 Implementation details

The website is written in the Python programming language using the ‘Flask’ framework. Configuration of the website is driven by a YAML format file that points to Salmon v0.12.0 output along with details like methods descriptions, UCSC track hub colors, bar plot colors, and any other experiment‐specific configuration. This greatly streamlines the process of adding new studies and new species. Data were processed using lcdb‐wf (https://github.com/lcdb/lcdb‐wf), which is itself driven by YAML configuration in a species‐agnostic manner, allowing for uniform processing across all studies.

## 3 RESULTS

**Figure 1:**
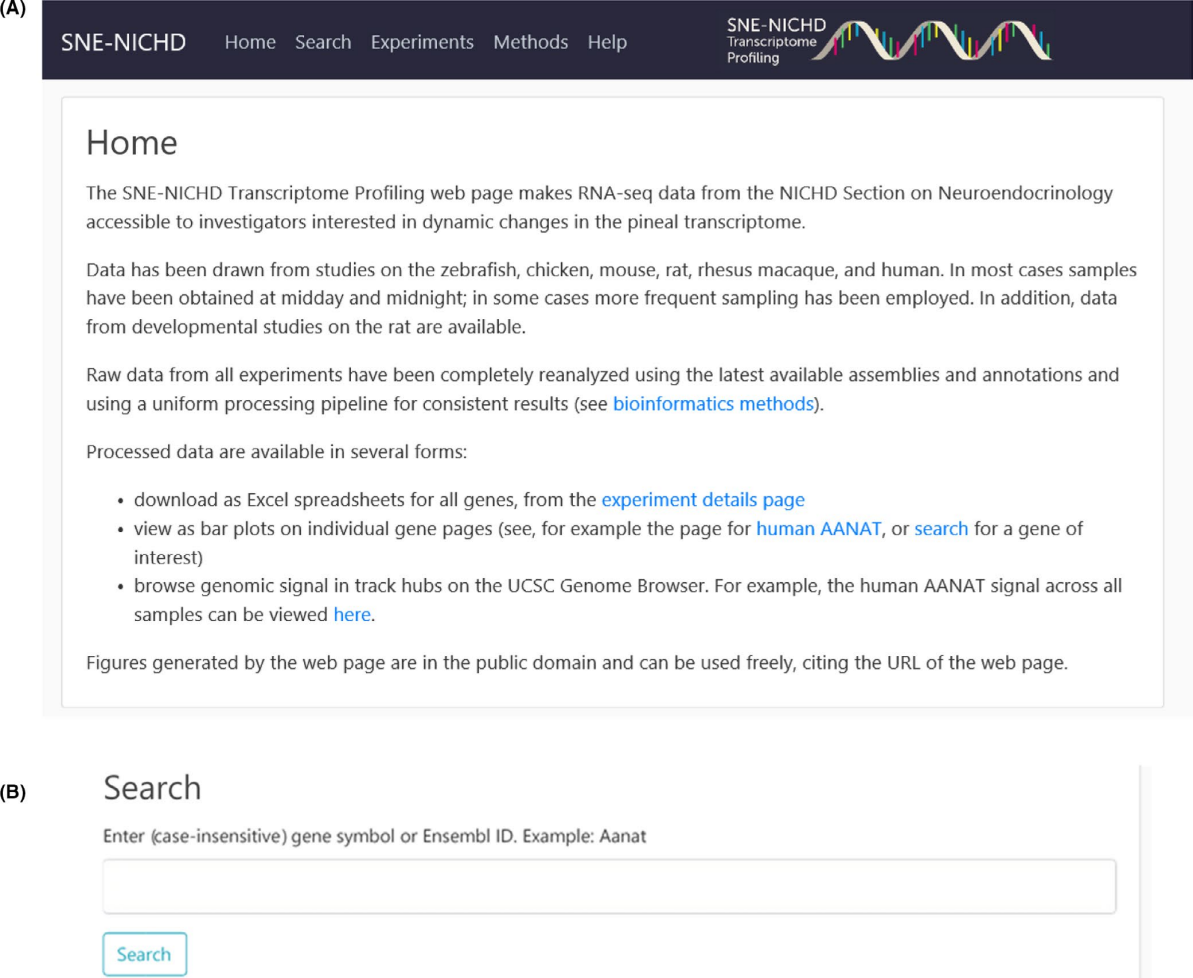
Home page and Search subpage. A, The Home page (https://snengs.nichd.nih.gov/home) opens subpages which are organized to search for genes specifically and to retrieve information relevant to the Experiments and datasets. In addition, the Methods page contains useful information about the Bioinformatics methods and the Help page has useful videos on use of the UCSC Genome Browser. B, The Search page (https://snengs.nichd.nih.gov/search). Entering an official gene symbol or an Ensemble ID symbol in the Search box retrieves data from all species. For aliases please refer to the Ensembl or NCBI Gene databases

**Figure 2:**
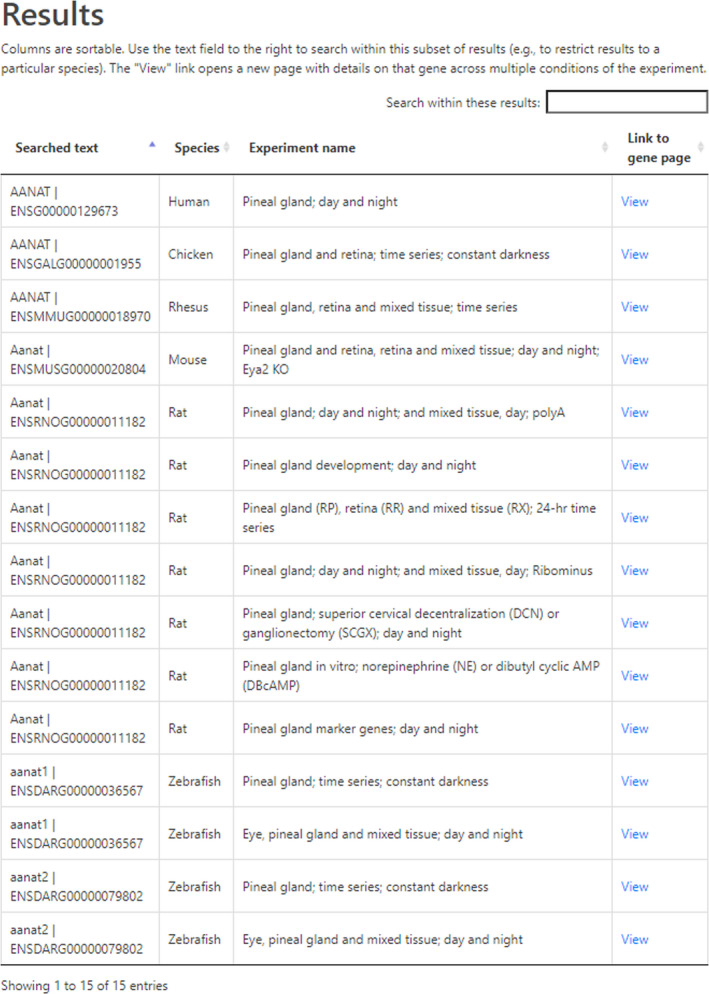
Results subpage. The results of querying a gene symbol generates a listing of the experiments and species in which the gene was found (https://snengs.nichd.nih.gov/search). Depending on the size of the gene family, multiple gene symbols may be displayed. In this case, one has to use the data cautiously. From this page, highlighted links (View) direct the user to the Gene subpage, which lists results of a single species and experiment

The Home Page (https://snengs.nichd.nih.gov) introduces the user to the main sections of the database (Figure 1A). Selecting the Search section displays the Search subpage (Figure 1B). Entering a gene symbol (*ie,* Aanat) in the query box opens the Results subpage, which contains a listing of species and experiments (Figure 2). The Search function will accept alternative symbols; however, when difficulty is encountered obtaining a result, the user is encouraged to refer to gene databases for assistance. This page contains information on the samples, including species, a brief description of the experiment and a Link to the gene page.

**Figure 3:**
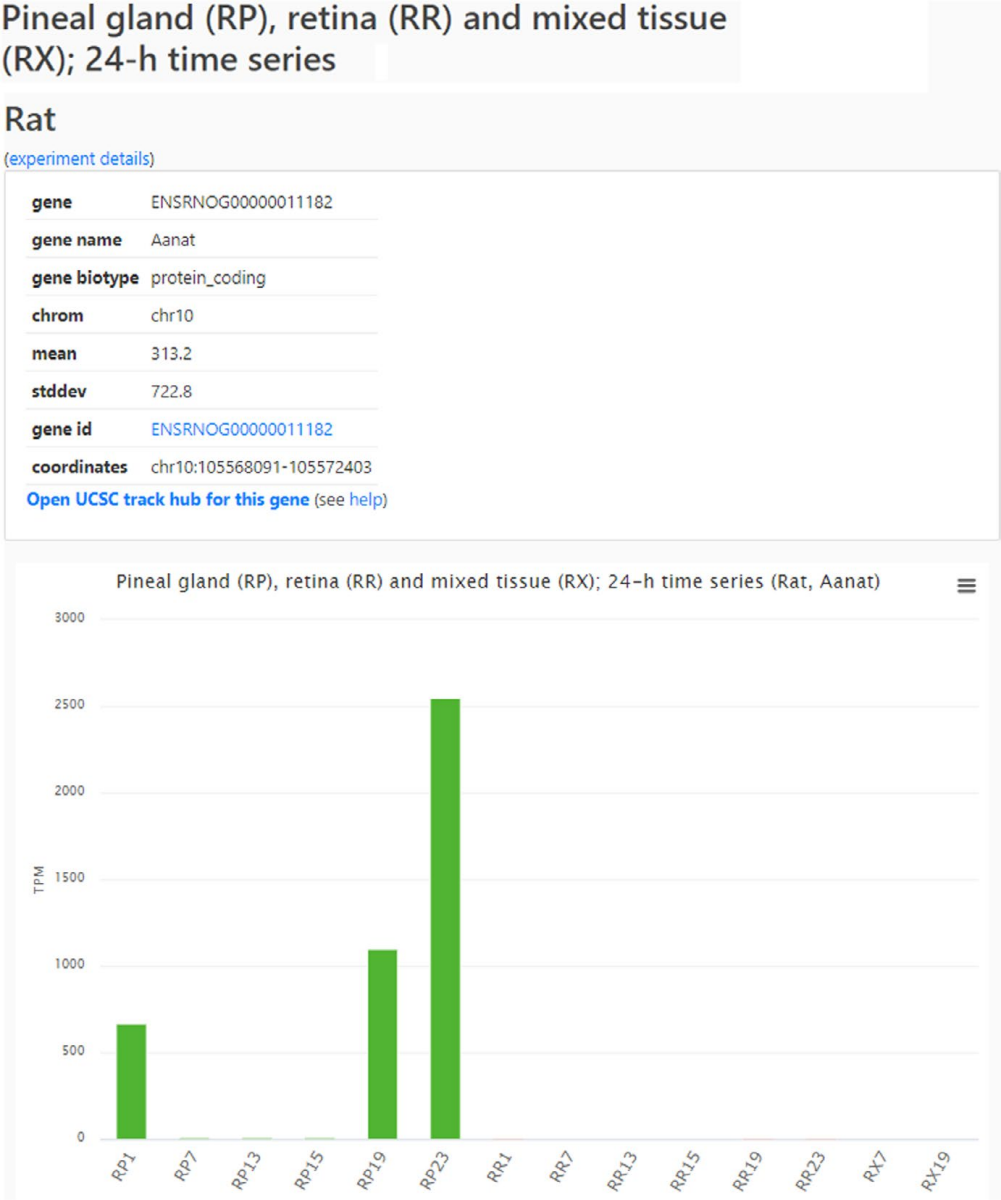
Gene subpage. An example of the gene page (https://snengs.nichd.nih.gov/species/Rat/gene/ENSRNOG00000011182#Time_Series‐anchor) that displays information about a specific gene including relevant experimental details, the UCSC track hub (Open UCSC track hub for this gene) and a help page for use of the UCSC Genome Browser. General information about each gene is available by clicking on the gene id, for example, ENSRNOG00000011182. Experimental results are displayed below in a bar graph. In addition, accessing the results of a single experiment will open other experiments dependent on availability

Clicking on that Link (Figure 2), opens a Gene subpage (Figure 3) with links to the Ensembl data (gene id) and the UCSC Genome Browser for the gene (Open UCSC track hub for this gene), in addition to presenting experimental results in a bar graph. These results are normalized count data (in TPM). In cases where multiple experiments for a species exist, all experiments are displayed and can be viewed by scrolling vertically.

**Figure 4:**
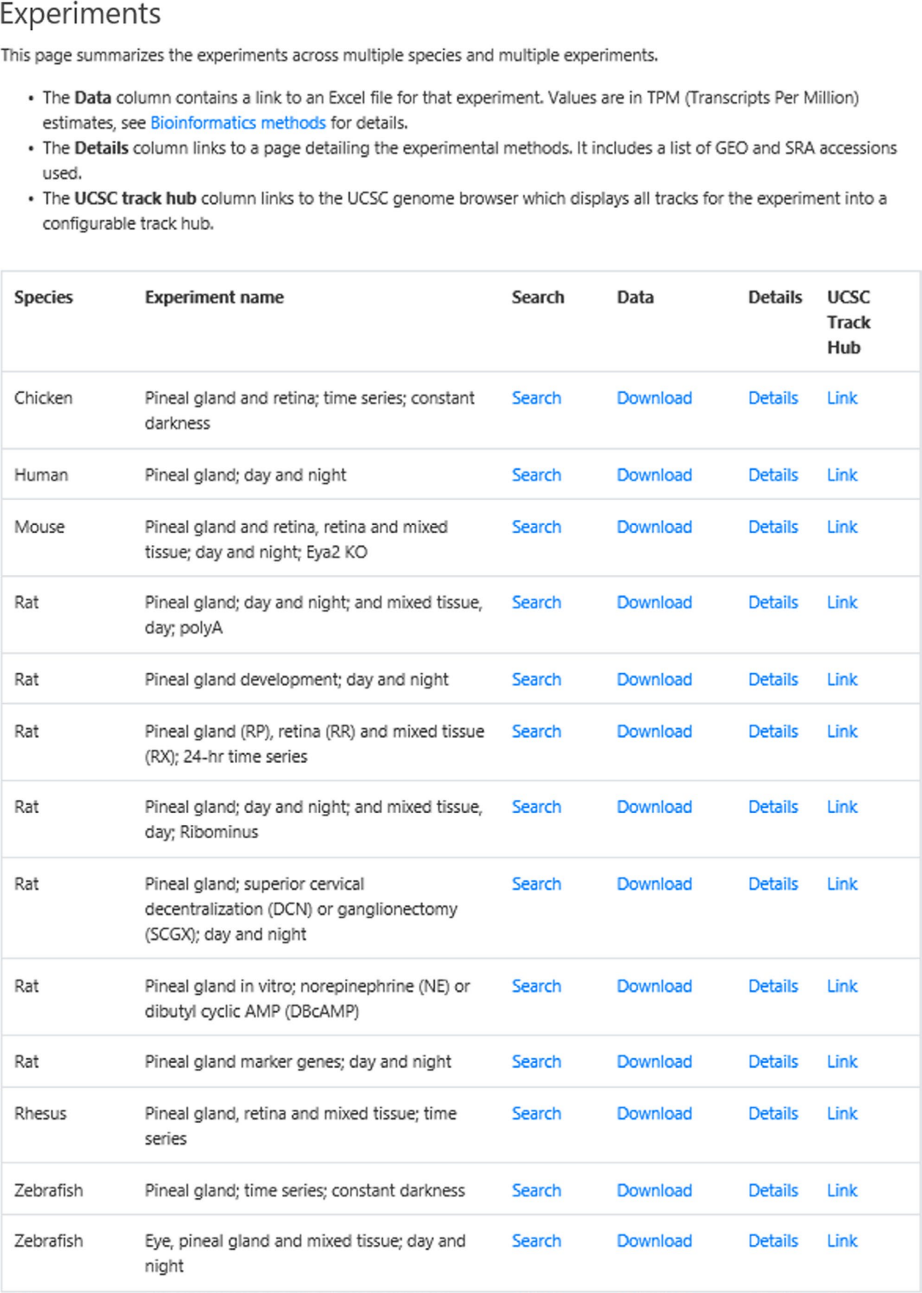
Experiments subpage. The Experiments subpage (https://snengs.nichd.nih.gov/experiments) is accessed from the Home page. It is an index for all experiments, leading to several resources. The highlighted link retrieves the Search subpage, described above. The Data link (Download) returns the data for an entire experiment in an Excel file, which also contains the average expression values and variance. Selecting Details opens the page with experimental details (see Figure 5). The highlighted link to the UCSC Track Hub (Link) gives access to the mapped data on the UCSC Genome Browser

**Figure 5:**
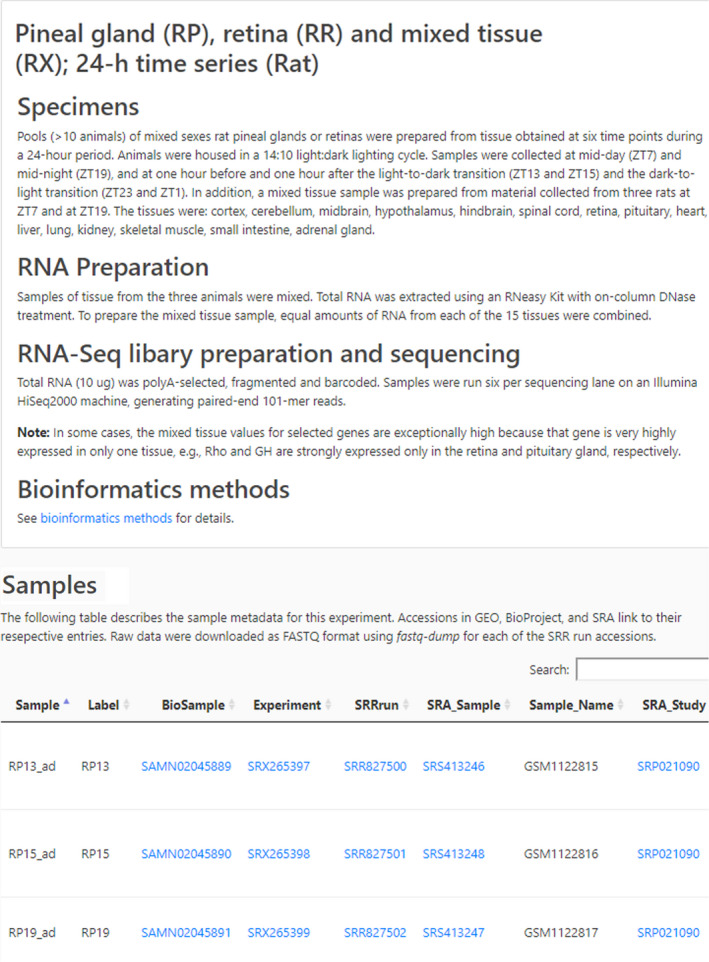
Details subpages. The Details subpages are accessed from the Experiments subpage (see Figure 4; https://snengs.nichd.nih.gov/experiments) by clicking on Details for a specific experiment. This yields information on sample preparation, RNA preparation, and data processing; and, the location of archived data. The search box is used to interrogate the table with identifiers (eg, SRX3229487) or fragments of identifiers (eg, _04h) in the table. The Samples section in this Figure is truncated for presentation purposes

Selecting Experiments from the Home page displays the experiments with four links (Figure 4). The first is the Search subpage, described above. The second (Download) retrieves the data in an Excel file. The third retrieves the Details (Figure 5) of the experiment, including sample preparation and data analysis; scrolling horizontally is necessary to open the table. The last is a Link to the UCSC Genome Browser, which documents the location of reads mapping for each gene.

The Methods and Help pages are not presented as figures. The Methods page contains general information on the Bioinformatics methods and identifies the genomic assemblies used; the Help subpage has links to tutorials on the use of the UCSC browser and contact information for further assistance.

As an example of the utility of comparing data across multiple species in a uniform format, we searched for differences in the day/night levels of transcripts among species. As shown in Table 2, the large night/day rhythms in the transcript abundance of several genes in the rat are not seen in the rhesus monkey or to a similar degree in other species (https://snengs.nichd.nih.gov/search). This emphasizes the importance of post‐translational modifications that occur.^27^, ^28^ It also is a caution against making generalities based on studies of one species.

**Table 2:** Comparative analysis of rhythmic transcript levels in the vertebrate pineal gland

Species	Night/Day		Day/Night	
	>30‐fold	3‐ to 30‐fold	>30‐fold	3‐ to 30‐fold
Chicken	Gos	Spcs1, Gnb3, Lbh, Lypla1, Prdm8, Aanat, Tph1, Am89a, Ckmt1a, Chga, Ddc, Ndrg		Rbp4, Rcan2, SSx2ip, Chgb, Calb2, R3hdml, Atoh8, Efr3a
Human		DUSP1, HKDC1		
Mouse	Gh, Prl	Aanat, Odc1, Mat2a, Kif5c, Nap1l5, Tbc1d15, Crem, Tbc1d1, Tjap1, Ndufa3, Syt4, Mitf, Rmdn3, Extl3, Amd1, Ywhaz, Ccnl1, Slc3a2, Impa1, Azin1, Prosc, Iqcb1, Crx, Rab3gap1, Srxn1, Manf, Ppa2, Gja1, Psme2, Arf, Cbx7, Tph1, mt‐Ts2, Fgf12, Mpp6, Gnai2, Necap1, Tpm4, Atp2a2, Hdhd3, Rnf13, Ip6k1, Dnajb6, Sik3, Ergic1, Tmem229b, Clptm1, Hsph1, Auh, mt‐Tw, mt‐Ti	Igkj4, Igkj1, Tpt1‐ps3, Ighj4	Enpp2, Ttr, Chmp1a, Unc119, Ccnd2, Acp2, Atp6v0a2, Tef, Igf2. Ermard, Lamb2, Fabp7, Twf1, Ewsr1, Etf1, Fxyd1, Arih2, Zfand6, Wbscr22, Ndrg1, Tbc1d17, Cox17, Fam166a, Atox1, Rpgrip1, Ackr1, mt‐Tl1, Dpysl3, Cisd3, Prpf19, Sag, Tpm3, Ift46, Apod, Taz
Rat	Aanat, Atp7b, Slc15a1, Dclk3	Irs2, Crem, Sik1, Ptch1, Cd24, Zrsr1, Rcan1, Kctd3, Bsx, Mat2a, Etnk1, Camk1g, Mbnl2, Gxylt1,Gem, Nptx1, Pcdh1, Eml5, Galnt16, Pde4b, Reep2, Syt4, Tjp2, Snap25, Hbb, Hba‐a2, Dnm2, Fkbp5, Man2a1, Fry, Dclk1, Mcam, Arhgap24, Hspa5, Slc17a6, Farp2, Rhob, Cry2, Lamb1, Hsph1, Ncald, Abca1, Mapk6, Ankrd52, Snrk, Slc7a6, Shroom3, Sik2,Ttc8, Nacad, Qsox1, Xpot, Zhx1, Wipf3, Abcf1,Frmpd1	Matr3	Gucy1a1, Frmd4b, Eef1a2, Scrn1, Hook1, Ttr, Pdc, Cfl2
Rhesus		PENK, CCN2, RP1, FAM167a, TGFBR3, ATP2A3,		OPN1SW, VASH1, PDC, GNGT1, LMOD1
Zebrafish	Nr1d1	Sik1, Dbpb, Dusp1, Dtx4, Rdh8b, Gjd2b, Gpr137bb, Aanat2, CR391986.1, Dclk2a, Guca1a, Tph1a, Gchi1, Ptn, Myh9a, Lpde6ga, Id2b, Cxcl14, Gabarapb	Nfil3‐5	Bhlhe40, Rbp3, Cry1aa, Rorcb, Pde6ha, Rorca, Camk1gb, Rbp4, Rp4l, Kera, Ry1bb, Per2, Irbpl, Cyp27c1, Nfil3‐6, Pfkfb4b, Sagb, Ahcy, Sdha, Eno1a, Add45ga, Tmtops2a, lrp1a, Hbba1, Aldocb, Tmem237b, Gpr146, Aldoa, Jag2b, Aclya, Cry1ba, Ybx1, Rcvrn3, Acadm. Stra6, Hbba1

Note

The day and night levels of transcripts were compared by calculating night/day and day/night ratios of normalized values (TPM + 0.1). Noncoding RNAs were eliminated. Only the top 1000 genes with official symbols were further grouped by ratios into greater than 30‐fold and 3‐ to 30‐fold differences. Genes are listed according to strength of rhythm. Human pineal data are included, noting that times of death and of tissue removal were not tightly controlled; accordingly, the indication of rhythmicity might be impacted. The data were downloaded from the Experiments page. In addition to the single datasets for chicken, human, mouse, and rhesus, the zebrafish “eye, pineal gland & mixed tissue” and rat “pineal marker genes” datasets were used.

The data also focus on the similarity of the genomic profiles of pineal glands from the species studied (Table 3). Selective expression of each gene was calculated as the ratio of expression of a specific gene in the pineal gland to that in a mixture of RNA from a group of tissues. As expected, three genes responsible for melatonin synthesis (Tph1, Aanat and Asmt) were selectively expressed in the glands studied. Another group of genes selectively expressed in the pineal gland includes those established as markers of the retina. The high expression of these genes only in the pineal gland and retina is known.^21^, ^22^ However, the specific functions of these retina‐related genes and other selectively expressed genes in the pineal gland have not received significant attention and deserve further analysis.

**Table 3:** Highly conserved selectively expressed pineal transcripts

	Genes selectively expressed among top 1000 genes
Three species	Adra1a, Adrb1, Ankrd33, Casz1, Drd4, Gngt*, Grk*, Grm*, Guca1a, Impg1*, Kif*, Opn*, Pax3, Pcdh*, Pla2g*, Ppef2, Prph2, Rdh*, Rp1*, Rps*, Rxrg, Slc16*, Slc6a*, Trim*
Four species	Aanat*, Aipl1, Asmt, Bsx, Cabp*, Cacna1*, Cacna2d*, Celf3, Chrna3*, Chrnb4, Cngb3*, Col*, Cplx*, Crb*, Crx, Gch1, Impg2, Isl2, Kcn*, Lhx4, Lrit*, Myo*, Neurod*, Otx2, Pde6*, Ptprn, Rbp3, Slc24*, Slc38a*, Tmem*, Tph1, Ush2a

Note

Genes were ranked according to selective expression in the pineal glands from zebrafish, mouse, rat, and rhesus. Expression was normalized (TPM + 0.1) and selective expression was calculated relative to expression in a mixture of tissue. The top 1000 selectively expressed genes were identified and those present in three or four out of four of the species are listed above. The data sources are given in the legend to Table 2. Asterisk (*), more than one homolog exists in some species; for example, Aanat* represents Aanat in mouse, rat, and rhesus in addition to Aanat1 and Aanat2 in zebrafish.

An analysis of the conserved highly expressed and tissue specific transcripts in the pineal gland, retina and in both tissues (Table 4) was done by identifying the highly tissue specific transcripts. They were then binned according to their expression ratio (pineal gland: retina). The results reveal a relatively smaller sets of pineal‐specific and retina‐specific genes, and a larger group of genes expressed in both tissues. Noting that these genes are selectively expressed only in these two tissues and not in others, it is highly likely that these genes represent evolutionarily conserved elements that can be considered to be related to the common origin of both tissues. In some cases, their roles have been identified, but in many cases, a functional role has not been established.

**Table 4:** Transcripts enriched in pineal gland, retina, and both the pineal gland and retina

Group	Enriched transcripts	
	Four of four species	Three of four species
Pineal gland	Aanat*, Asmt, Chrnb4, Gch1, Gnat2, Gnb3*, Guca1a, Lhx4, Pde6c, Sall1*, Tph1*, Pax3	Alx4, Bsx, Chrnb3, Gngt2*, Lrrc38, Ptpn20
Pineal gland and retina	Arr3*, Cabp4, Cacna1f*, Cacna2d4, Cnga1*, Cngb3, Cplx4*, Crb2*, Crx, Drd4, Fam161a, Gabrr1, Gabrr3*, Gngt1, Grk1*, Guca1b, Impg1*, Impg2*, Kcnb*, Lrit1*, Mpp4, Msi1, Myo*, Nyx, Opn1sw, Otx2*, Pdc*, Pde6g*, Rbp3, Rlbp1, Rom1*, Rp1l1, Slc24a1, Stx3, Tulp*, Unc119*, Ush2a	Adrb1, Cabp5* Crabp*, Crb1, Crocc, Egflam, Fabp*, Fam169a, Gnb5, Gng1, Grik1*, Gucy2d, Hcn*, Igsf9, Impdh1, Kcn*, Kcna*, Kcnj14, Lrit2, Lrit3, Mak, Mgarp, Neurod4, Ntng2, Nxnl1, Pcdh15, Pla2g*, Plch2, Ppef2, Prph2*, Prss3*, Rax*, Reep6, Rorb, Rrp1b, Samd11, Slc16a*, Slc17a*, Slc24*, Slc38a*, Slc39*, Slc4*, Slc6a6, Tmem215, Tmem237*, Trpm1*
Retina	Abca4*, Ankrd33*, Ccdc*, Cdhr1, Chrna3a, Col*, Cryaa, Fscn2, Gucy2f, Irs1*, Isl1, Kcnv2*, Nr2e3, Nrl, Pde6a, Pde6b, Pde6h, Rdh8, Rho, Rpl, Rrh, Sag*, Sh2d*, Six3*, Slc1a7, Tfap2*, Vsx1, Vsx2	Cryba*, Crybb2, Crygm*, Gabrr2, Glb1l2, Gnat1, Grm6, Isl2, Lgsn, Lim2, Mab21l1, Opn1mw*, Opn4*, Pax6*, Prdm13, Prph*, Rcvrn*, Rgr, Rtbdn*, Samd7, Vax2

Note

Enrichments in the pineal gland and retina relative to other tissues have been assessed by the determining the ratio of the normalized (TPM ++.1) abundance of a transcript in each tissue relative to that in the mixed RNA sample (TPM + 0.1) to yield a relative expression value (rEx). Mixed RNA samples were made by mixing equal amounts of RNA from 6 to 20 tissues. The rEx values of the top 300 enriched transcript from the pineal gland and the top 300 from the retina were compared (pineal gland rEx/retina rEx) and transcripts that were > 10‐fold were binned as pineal gland and those < 10 fold as retina; maximum levels were approximately 1000 for the pineal gland and 1/1000 for the retina. The remaining transcripts comprise the pineal gland and retina group. Zebrafish, mouse, rat, and rhesus are included. The rat data are from the 24‐hr time series experiment, the zebrafish data from the experiment with mixed tissue, and the rhesus and mouse data are from single experiments; the latter is from 129sv mice. Data from all time points have been averaged and normalized (TPN + 0.1). The results above indicate whether a listed transcript is detected in all four or in only three species evaluated. Asterisk (*), more than one homolog exists in some species; for example, Aanat* represents Aanat in mouse, rat, and rhesus in addition to Aanat1 and Aanat2 in zebrafish.

## 4 DISCUSSION

This database will serve as a foundation for future molecular biological research on the pineal gland and retina, making available the data to scientists with a computer and an internet connection. The uniform processing of raw data makes the comparison of results more meaningful and takes advantage of advances in tools, algorithms, assemblies, and annotations since original publication. Whereas the human and mouse genomes are the most highly annotated, and the chicken and zebrafish less so, the maturity of all annotations allows for in‐depth analysis of nearly all genes. A potential problem is that symbols used for identification of a gene in one species may not be used in other species or may be used for different genes. Hence, in cases where identification is questionable, confirmation may require analysis of sequence homology.

### 4.1 Utility and accuracy of RNA‐Seq data

In judging the utility and accuracy of the RNA‐Seq data, it should be noted that there is good agreement with data from other methods for the analysis of pineal gland and retina material, including microarray, Northern blot and qRT‐PCR as regards day/night differences.^5^, ^6^, ^7^, ^8^, ^9^, ^10^, ^11^, ^12^, ^13^ Accordingly, the RNA‐Seq data can be viewed as highly useful and reliable.

The method also has advantages over other methods, perhaps the most important is that it sequences all transcripts, including those without a history in any literature. This opens new avenues for study for the pineal gland and retina. One of the most fertile areas is the identification of noncoding RNAs, both micro RNAs and long noncoding RNAs.^9^, ^23^ Some of these are known to have daily rhythms in both tissues. Noteworthy is the discovery of a unique micro RNA‐183‐96‐182 cluster in the pineal gland and retina,^9^, ^29^ which represents the major component of pineal miRNAs. Accordingly, it can be considered to be an additional marker of the common ancestral photodetector which gave rise to the pineal gland and retina. Although the function of this cluster remains unknown in the pineal gland, it has been reported to play a role in phototransduction and development in the eye.^30^, ^31^

Study of pineal miRNAs also led to the discovery of very high levels of pY RNA1‐s2 in the retina, relative to other tissues,^25^ including the pineal gland. Moreover, it was found that pY RNA1‐s2 selectively binds the nuclear matrix protein Matrin 3 and to a lesser degree to heterogeneous nuclear ribonucleoprotein U‐like protein. The distribution of pY RNA1‐s2 in all retinae and retinal cell lines suggests a role in vision. Both these discoveries could not have been made using methods other than RNA‐Seq.

Likewise, the finding of robust daily rhythms in the abundance of several long noncoding RNAs^23^ in the pineal gland under neural control, and the discovery of expression of lncSN134 in both the retina and pineal gland was dependent on the use of RNA‐Seq. The long noncoding RNAs range in size significantly and like pineal miRNAs, remain largely unstudied and unknown.

Whereas RNA‐Seq is a powerful technique, the results must be viewed with healthy skepticism, especially with transcripts that are weakly expressed and when evaluating small night/day differences in transcript abundance. Confirmation by an independent method should be considered. In addition, in the case of weakly expressed transcripts, the mapping of reads on the UCSC browser should be evaluated to confirm that the read assignment pattern is consistent with the intron/exon features of the transcript.

### 4.2 Experimental design

A problem that is considered in any study designed to measure day/night differences is the number of time points per day. Often this is limited by factors including the housing of animals and the number of animals per point necessary to obtain sound data. RNA‐Seq introduces another factor, the cost of analysis. Accordingly, the design of the studies included in the database (Table 1) is also a reflection of the cost of sequencing and bioinformatics. The studies included sampling that ranged from two to six time points per day. When sampling is done at only two time points, noon, and midnight, the potential for overlooking a dawn/dusk rhythm exists. Accordingly, it is best not to limit experiments to two time point studies and to use four or more to detect daily rhythms. However, in the case of study of daily rhythms in the pineal gland, a two time point study will capture most large changes. Moreover, this approach is highly instructive, in that it provides valuable data on the levels of tens of thousands of transcripts. Accordingly, one can see merit in such studies.

The number of replicates to use is also another important issue. RNA‐Seq data are typically highly reproducible for most transcripts when normalized. This reflects a feature of the method, in that there is redundancy in the detection of a transcript, as a result of fragmentation and amplification. In the final analysis, each calculated transcript level is not simply a single measurement, but reflects multiple detection events, depending on the size of the transcript and abundance. Accordingly, in N = 1 situations, it is possible to obtain an indication of statistical variance of all transcripts, and use this to determine whether, for example, a day/night difference is statistically significant.

### 4.3 Transcriptomics versus proteomics

Whereas RNA‐Seq does provide a highly useful tool for the discovery and characterization of transcripts, it is not a substitute for proteomics. The study of an mRNA and its encoded protein often are in agreement as regards the presence and dynamic changes in both. However, this is clearly not the case in all situations.

An excellent example is Aanat. In the rat, Aanat mRNA, protein, and activity increase at night, reflecting phosphorylation of the protein at two sites. When lights are turned on in the middle of the night, a rapid decrease in enzyme activity occurs, with little change in mRNA levels. The changes in enzyme activity are due to dephosphorylation of the protein, which is rapidly destroyed by proteasomal proteolysis, as reviewed.^32^ Another example of mRNA levels and protein levels not exhibiting similar dynamics is found in studies of the rhesus pineal gland. There is little daily change in mRNA encoding Aanat, although the changes in enzyme activity are robust.^33^ These observations are evidence that it is necessary to determine whether changes in an mRNA are associated with changes in an encoded protein to determine the relationship. Unfortunately, the science of proteomics has not advanced to the all‐inclusive nature of mRNA analysis, in part because it is difficult to uniformly detect the possible post‐translational modifications.

Use of the database will allow investigators to initiate efforts to identify transcripts that are highly expressed in the pineal gland relative to the retina and or other tissues, transcripts that are highly expressed in the pineal gland of one species but not another, transcripts that exhibit marked night/day differences, transcripts that are under neural/adrenergic cyclic AMP control, and transcripts that exhibit changes in expression during development. In doing so, the web page should promote and enhance future studies of pineal cell biology.

### 4.4 Referencing the web page

The data on the web page are in the public domain and the use of the figures and data does not require authorization of the authors. The web page should be referenced by citing this publication.

## CONFLICTS OF INTEREST

No conflicts of interest related to this manuscript exist.
